# ADAPTATIONS IN A DRUMMING AND HORTICULTURE INTERVENTION FOR OLDER PEOPLE LIVING WITH INTELLECTUAL DISABILITIES

**DOI:** 10.1093/geroni/igae098.2240

**Published:** 2024-12-31

**Authors:** Emily Ihara, Michelle Hand

**Affiliations:** George Mason University, Fairfax, Virginia, United States; George Mason University, Fairfax, Virginia, United States

## Abstract

People aging with intellectual and development disabilities (IDD) often experience intersectional discrimination, impacting multiple dimensions of wellness. Rhythm & Nature is a trauma-informed drumming and horticulture intervention that provides an inclusive and modifiable environment. Both activities involve bilateral stimulation, using alternating hand movements to help create connections between the left and right hemispheres of the brain, which can help improve anxiety symptoms, memory, creativity, and attention. The 8-week intervention was delivered two times, roughly one year apart. The convenience sample was drawn from attendees of an adult day center for older people living with IDD, who could participate in drumming, horticulture, or both activities. This presentation will provide qualitative analysis results of the eight participants in Group 1 and preliminary analysis of the 18 participants in Group 2. Themes from the Group 1 analysis included autonomy, voice, and creativity; fun, laughter, and joy; safe space for sharing; and skill development, affirmations, and confidence. The acuity level of Group 2 participants was higher and the group was larger, requiring different modifications by the two facilitators. Adaptations that improved accessibility included formatting the emojis differently on the pre/post forms, printing pre/post surveys on different colored paper to make it easier for them to follow, and focusing on one theme per session. Week 4 of Group 2 occurred during the center director’s last day. Participants were able to drum a farewell song and create a bouquet to convey meaningful goodbye messages. Further program examples and implications for research and practice will be provided.

